# Effects of Active Oxygen Toothpaste in Supragingival Biofilm Reduction: A Randomized Controlled Clinical Trial

**DOI:** 10.1155/2019/3938214

**Published:** 2019-07-01

**Authors:** Emanuelle Juliana Cunha, Caroline Moreira Auersvald, Tatiana Miranda Deliberador, Carla Castiglia Gonzaga, Fernando Luis Esteban Florez, Gisele Maria Correr, Carmen Lucia M. Storrer

**Affiliations:** ^1^Graduate Program in Dentistry, Universidade Positivo, Rua Professor Pedro Viriato Parigot de Souza, 5300, Cidade Industrial, Curitiba, PR, Brazil; ^2^The University of Oklahoma Health Sciences Center College of Dentistry, Division of Dental Biomaterials, 1201 N. Stonewall Avenue, Room 146B, Oklahoma City, OK 73117, USA

## Abstract

Gingivitis is still considered a major risk factor for the occurrence and progression of periodontal disease. The aim of the present study was to compare the long-term (1, 12, and 18 weeks) antiplaque and antigingivitis efficacies of two commercially available toothpastes, Colgate Total^®^ (CT) and BlueM^®^ (BM), against attached supragingival dental plaque and gingival inflammation in an experimental gingivitis model. A parallel double-blinded randomized clinical trial including 39 dental students who refrained from all plaque control methods (manual or chemical) for 7 days was conducted. After the establishment of clinical gingivitis, participants were randomized into two experimental groups (CT and BM). Plaque index (PI) and gingival index (GI) were then calculated according to Turesky's modified Quigley and Hein index. Participants were assessed in four time periods (preclinical trial phase (W −1), gingivitis phase (W0), one week (W1), twelve weeks (W12), and eighteen weeks (W18)). Participants' stimulated saliva was collected and cultured (either aerobically or anaerobically, 37°C, 48 hours) in each time period (W −1, W0, W1, W12, and W18) for the count of viable colonies. Obtained data were analyzed using 2-way ANOVA and Tukey's test (*α* = 0.05). No significant differences were found (*p* > 0.05) between experimental groups at W −1. Significant differences between groups were observed at W0 (*p* < 0.05) for the parameter time period, but not for the interaction between parameters (time period *∗* toothpastes). Lower bacterial counts were observed in both groups after one week of toothbrushing; however, no significant differences were found between investigated dentifrices. Intra- and intergroup comparisons revealed that significant differences were not found (*p* > 0.05) between dentifrices at W1, W12, and W18 for both GI and PI. The present study demonstrated that toothpastes containing active oxygen and lactoferrin (BM) have comparable antiplaque and antigingivitis efficacies with triclosan-containing toothpastes (CT).

## 1. Introduction

Gingivitis is a very common type of soft tissue inflammation. Despite all efforts from the scientific and manufacturing communities, this condition continues to be considered a major risk factor for the establishment and progression of periodontal disease and tooth loss [1]. Patient orientation and implementation of meticulous oral hygiene techniques based on the utilization of toothbrushes (traditional or interdental), dental floss, and antiplaque fluoride-containing toothpastes is typically the first conservative approach used to treat patients with such an inflammatory condition [2]. Professional in-office mechanical techniques (manual or rotary) are based on debridement of tenaciously attached dental plaque and polishing of the clinical crown to not only remove biofilms but also reduce the roughness (*R*_a_) of biotic (enamel, dentin, and cementum) or abiotic (metals, polymers, and ceramics) saliva-coated surfaces within the oral cavity.

A recent systematic review of the literature investigated the impact of dentifrice formulation on the ability of these materials to prevent biofilm attachment and regrowth. The results reported have indicated that dentifrices containing fluoride and no active antiplaque ingredients in their formulations displayed only weak inhibitory effects against the regrowth of oral biofilms when compared to water or saline solutions [3]. In this context, manufacturers have incorporated several active ingredients (stannous fluoride (SnF), triclosan (Tcs), and activated edathamil) [4–6] to commercially available dentifrices to improve their surface-active properties against oral biofilms. Previous studies have demonstrated that both SnF and Tcs were effective agents to control and reduce dental plaque [7, 8] when compared to dentifrices containing only fluoride in their compositions. The former agent has been correlated with relevant antimicrobial and anti-inflammatory effects based on Tcs' ability to inhibit the cyclooxygenase and lipoxygenase pathways in the metabolism of arachidonic acid in a concentration-dependent manner.

Hydrogen peroxide (H_2_O_2_) is known for its ability to spontaneously dissociate into several reactive oxygen species (ROS), for its strong chemotaxis effects on leukocytes, for lipid peroxidation of bacterial cell walls, and for disturbing the neutrophil respiratory burst in wound fluids and, therefore, is considered a relevant broad range and nonspecific antibacterial agent [9]. Bluem International (Wapenveld, Netherlands) has capitalized on this conceptually feasible disinfection technology topic and recently introduced the BlueM® dentifrice. This over-the-counter toothpaste contains active oxygen and lactoferrin in its composition, and according to its manufacturer, the underlying mechanism of action is based on the controlled delivery of ROS to injury sites.

Despite these interesting claims put forth by the manufacturer, very little information is available in the literature regarding the antibacterial and local anti-inflammatory effects of this novel dentifrice and its utility in controlling the accumulation and growth of oral biofilms. Therefore, the main objective of the present study was to investigate the antibacterial and anti-inflammatory efficacies of two commercially available dentifrices with distinct mechanisms of action containing either Tcs or active oxygen and lactoferrin in an experimental gingivitis model for 1, 12, and 18 weeks. The working hypothesis tested was that dentifrices containing active oxygen and lactoferrin were more antibacterial and anti-inflammatory than Tcs-containing products.

## 2. Materials and Methods

The present parallel and double-blinded randomized clinical trial was approved by the Ethics Committee of Universidade Positivo (CAAE: 55280416.1.0000.0093) and was registered in ReBEC (RBR-48MVDX). The present study was based on an experimental gingivitis model proposed by Löe et al. [10].

### 2.1. Participants and Inclusion and Exclusion Criteria

First-year students from the Universidade Positivo College of Dentistry who volunteered to participate in the present study were recruited and triaged to determine their eligibility to participate in the present study. The inclusion criteria were age between 18 and 35 years; a minimum of 28 teeth; absence of caries; presence of preestablished gingivitis (gingival index (GI) ≥ 1 and plaque index (PI) ≥ 1) [11, 12]; probing depths and attachment loss smaller than 3.00 and 2.00 mm, respectively; willingness to participate in the present study; and overall good health. The exclusion criteria included antibiotic therapy 3 months prior to participating in the present study, presence of removable or fixed orthodontic appliances, impaired motor skills, smokers, and lack of compliance with study guidelines. Individuals successfully matching the inclusion criteria received a soft flat bristled toothbrush (Colgate-Palmolive Company) and detailed explanations (oral and written) regarding the objectives, the experimental protocols, and the requirement to not brush their teeth for seven days to establish clinical gingivitis [10, 13]. After that, participants were requested to sign a form to give consent and to demonstrate adherence and compliance to the guidelines of the present study.

### 2.2. Sample Size

For the primary parameter PI, a mean difference Δ of 0.25 between test and control and an assumed standard deviation *σ* of 0.30 were chosen. A sample size of 18 per group was calculated with a power of 80% and a significance level of 95% (http://www.lee.dante.br).

### 2.3. Gingival Index (GI) and Plaque Index (PI) Determination

The determination of the GI and PI was performed using modified versions of the protocols published by Löe [11] and Turesky et al. [12], respectively. The clinical parameters of interest were applied to all teeth except for third molars. Gingival tissues adjacent to each tooth surface were scored as follows: 0 = absence of inflammation; 1 = mild inflammation with small change in color and texture; 2 = moderate inflammation including moderate glazing, redness, edema, and hypertrophy associated with bleeding on probing; and 3 = severe inflammation characterized by redness and hypertrophy displaying spontaneous bleeding.

Aliquots (10 mL/participant) of plaque-disclosing solution (Eviplac solution, Biodinamica-Ibiporã, PR, Brazil) were used according to the protocol previously published by Turesky et al. [12] that modified the classification firstly defined by Quigley and Hein to quantify dental plaque. Each tooth was divided into six regions ((1) mesiobuccal, (2) middle-buccal, (3) distobuccal, (4) mesiolingual, (5) middle-lingual, and (6) distolingual) to facilitate the plaque-scoring process. The amount of plaque observed on each surface was then scored as follows: no plaque (0), separate flecks or flecks of plaque at the cervical margin (1), thin continuous band of plaque (up to 1.0 mm) at the cervical margin (2), continuous band of plaque (>1.0 mm) covering less than 1/3 of the clinical crown (3), plaque covering between ≥1/3 and ≤2/3 of clinical crowns (4), and plaque covering ≥2/3 of the clinical crowns (5). The final GI and PI scores for each participant were then calculated by adding the scores of each tooth and dividing the obtained value by the number of teeth examined.

### 2.4. Intraexaminer Reproducibility Assessment

Two previously trained examiners (blinded to treatment allocation) were subjected to two independent sessions where they were calibrated regarding the assessment of both GI and PI. Dental students (*n* = 10) selected to participate in calibration sessions were not included in the present study to avoid examiners' unconscious bias. Examiners assessed each participating dental student for GI and PI. One hour later, examiners reassessed the students, and obtained results were statistically analyzed using the Cohen kappa test that indicated a high interexaminer reproducibility coefficient for GI (0.91) and PI (0.87), respectively.

### 2.5. Masking

All subjects were masked within their individual groups and received a group-specific kit of products containing one manual toothbrush and one toothpaste tube (either toothpaste “A” or toothpaste “B”). Toothpastes were allocated by simple randomization. Individuals pertaining to the Colgate Total (CT) group received a toothpaste tube identified as “A,” which contained 75 mL of Colgate Total^®^ (0.3% triclosan, 2% copolymer, and 0.243% (1,100 ppm) sodium fluoride; Colgate-Palmolive Company). Individuals pertaining to the BlueM (BM) group received a toothpaste tube identified as “B,” which contained 75 mL of BlueM^®^ (hydrogen peroxide, sodium perborate, honey, xylitol, and lactoferrin; Bluem International BV, Wapenveld, Netherlands). Toothpastes identified as either “A” or “B” were repacked by a contractor pharmacy, and details regarding toothpaste compositions were kept blinded to investigators until completion of the present study.

### 2.6. Study Design

**Figure 1:**
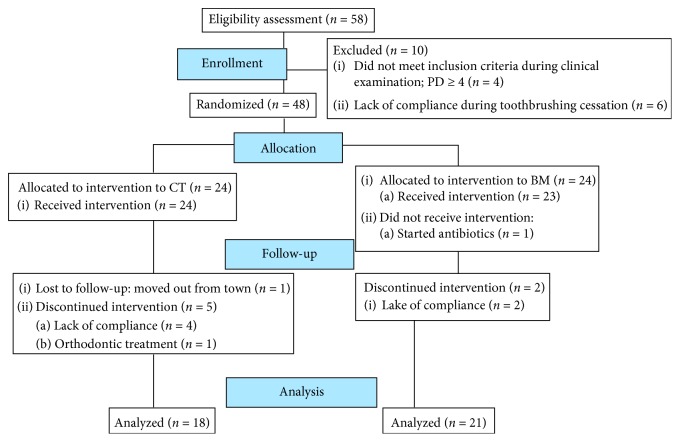
Flow diagram.

Figure 1 summarizes the outline of experimental procedures used in the present study. The study started with 58 students who volunteered and accepted the conditions previously explained. After that, a careful intraoral evaluation was carried out. Sterile Williams' periodontal probes (Millenium-Golgran, São Caetano do Sul, SP, Brazil) were used to measure the clinical parameters of interest (GI and PI). If during clinical examination participants met the inclusion criteria established, examiners would explain the objectives and guidelines of the present study. Participants that accepted the conditions and guidelines signed the informed consent and officially started to participate in the present study. All participants (*n* = 58) were then subjected to a professional dental prophylaxis to improve sample homogeneity. After that, participants were instructed to completely cease oral hygiene for seven days for plaque accumulation and development of an in situ gingivitis model. After seven days, participants were randomly assigned to one of the experimental groups (either CT or BM).

**Figure 2:**
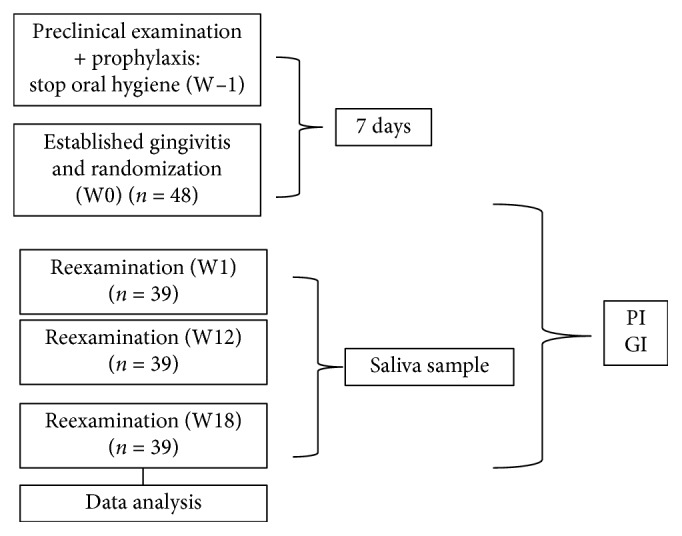
Experimental design.

Participants who had not developed clinical signs of gingivitis (<10% bleeding sites with probing depths ≤3 mm) [13] at seven days were excluded from the present study (*n* = 6). After that, participants (*n* = 48; 28 females and 20 males) received instructions regarding the modified Bass oral hygiene technique. Participants were then examined immediately after the establishment of gingivitis (W0) and after one (W1), twelve (W12), and eighteen (W18) weeks for PI and GI measurements (Figure 2). Each examiner scored participants' teeth in each clinical session. During each return, examiners verified participants' compliance by checking the utilization status of the toothpaste and toothbrush. The toothpaste (70 g = 75 mL) was given to the participants, and in each return, they were asked to bring the tube to check its weight and the toothbrush to check the appearance of the bristles, confirming the individual adherence to the study.

### 2.7. Microbiological Sampling

Aliquots of stimulated saliva (15 mL/patient/visit) were collected from participants of both experimental groups (CT and BM). Serial dilutions (10^5^) of saliva (aliquots of 100 *μ*l) were then cultured in 5% sheep blood agar plates (in both aerobic and anaerobic conditions) for 24 hours. Counts of total bacteria for aerobic and anaerobic conditions were obtained after 24 and 48 hours, respectively [14].

### 2.8. Statistical Analysis

Data on GI and PI were statistically analyzed using one-way ANOVA (toothpaste) with repeated measures and Tukey's test. Data on total bacterial count were analyzed using the nonparametric Mann–Whitney test because data were not normally distributed and to demonstrate the presence of significant differences between experimental groups. When necessary, comparisons were made by the Dunn test. All analyses were performed using the Statistical Package for Social Sciences software (SPSS version 20.0, Chicago, IL, USA) with a 95% confidence level (*p* < 0.05).

## 3. Results

Thirty-nine participants completed the 18-week follow-up period (22 females and 17 males). The compliance check revealed that 100% of these participants have used the amount of toothpaste expected for the investigation period. Tables 1 and 2 show the mean GI and PI values observed in both groups throughout the study. Results reported for GI have indicated the presence of significant differences for the factor “time” (*p* < 0.0001), wherein the results observed in W0 were significantly higher than those observed in the remaining time periods. Time periods (W1, W12, and W18), “toothpaste,” and the interaction between factors “toothpaste *∗* time” were demonstrated to be not significant predictors of treatment response (*p* > 0.05, *p*=0.8068, and *p*=0.750, respectively). Results reported for PI (primary factor) have indicated the presence of significant differences for the factor “time” (*p* < 0.0001), wherein the results observed in W0 were significantly higher than those observed in the remaining time periods. Time (W1, W12, and W18), “toothpaste,” and the interaction between factors “toothpaste *∗* time” were demonstrated to be not significant predictors of treatment response (*p* > 0.05, *p*=0.8068, and *p*=0.750, respectively).

At the preclinical trial period (W −1), before randomization, no statistically significant differences were observed between groups. A decrease in GI and PI mean values was observed immediately after the first week of observation (W1) when participants were brushing their teeth with either CT or BM; however, no statistical differences were observed between the experimental groups. Differences from intra- or intergroup comparisons for toothpastes (CT and BM) or time periods (1, 12, and 18 weeks) were found to be not statistically significant (*p* > 0.05) for both GI and PI.  Table 3 illustrates the time-dependent (W0–W18) results for total bacteria (CFU/mL) cultured in either aerobic or anaerobic conditions. The colony count results are shown in terms of median values (min–max), where it can be observed that bacteria cultured in aerobic conditions were associated with lower CFU/mL values when compared to bacteria grown under anaerobic conditions independently of the type of toothpaste or time period considered. It is also possible to observe that total bacterial counts significantly decreased after the utilization of oral hygiene techniques in a material-specific and a time-specific manner.

## 4. Discussion

The present parallel and double-blinded randomized clinical trial was designed to compare the antiplaque and antigingivitis efficacies of two commercially available toothpastes (CT and BM) and their positive impacts on gingival clinical parameters (GI and PI) over an 18-week follow-up period. At the end of the present study, no statistical differences were found between the two formulations for both GI and PI scores, thereby rejecting our working hypothesis. In order to assess the efficacies of the toothpastes investigated, participants received a standardized toothbrush and oral hygiene instructions prior to the preclinical time period (W0, no toothbrushing). At the end of the study, the total number of participants was 39 because some participants were excluded due to lack of compliance or due to personal reasons. The dropout rates (32.7%) reported in the present study are higher than those previously reported [15]. Such higher dropout rates can be explained by the population selected in the present study (dental students). On the contrary, participants' compliance during the toothbrushing phase (W1–W18) was 100%, as denoted by participants exchanging their empty dentifrice tubes for new tubes within the 18-week follow-up period.

In order to decrease initial periodontal variability, participants were selected from a population of dental students with presumably above-average oral hygiene techniques (toothbrushing and flossing). The GI and PI results reported for W0 (no toothbrushing period) suggest that even though our experimental design was based on a short period of toothbrush cessation, a clinical gingivitis model was established. These findings are corroborated by previous findings reported in the literature [13]. The GI (0.12 ± 0.16 and 0.08 ± 0.14) and PI (3.37 ± 0.55 and 3.45 ± 0.63) results at W1 have clearly shown that no statistically significant differences were found (*p* > 0.05) between experimental groups (CT and BM), which denotes that the population investigated was homogeneous.

Our findings have also demonstrated that significant differences between the antiplaque and antigingivitis efficacies of the two toothpastes investigated could not be observed (*p* > 0.05) independently of culture conditions (either aerobic or anaerobic) or time periods (1, 12 and 18 weeks) investigated, thereby rejecting the working hypothesis of the present study. Moreover, the results reported have demonstrated that both toothpastes investigated were effective in removing attached dental plaque and improving participants' oral health, as denoted by the active control of gingival inflammation. Even though these are clinically relevant and positive results, it has been previously demonstrated that Tcs (an active ingredient in CT) may bioaccumulate in humans (blood, breast milk, and urine) and may even adversely impact male fertility in a dose-dependent manner [16, 17]. Thus, BM may be considered a good alternative for the utilization of CT toothpastes because its utilization does not result in adverse effects and its active ingredients (oxygen and lactoferrin) were shown to be effective in removing attached dental plaque and controlling established gingivitis in an in situ human model, as presently reported. Lactoferrin, presented in the BM toothpaste, is a protein that possesses iron binding/transferring, antibacterial, antiviral, antifungal, anti-inflammatory, and anticarcinogenic properties [18]. A recent randomized clinical trial has demonstrated that a toothpaste containing enzymes and proteins was effective at preventing gingivitis compared to toothbrushing with a commercially available fluoride toothpaste [19].

Since participants had similar oral hygiene conditions at the beginning of the study and were subjected to professional prophylaxis prior to their participation, it is possible to affirm that the levels of plaque removal observed at W1, W12, and W18 were directly associated with the plaque removal efficacies of the toothpastes investigated. Nevertheless, toothbrushing with conventional manual techniques has been shown to not completely remove dental plaque. A systematic review recently published verified that mean plaque scores typically decrease approximately 42%, following a manual toothbrushing session [20], because of the brushing regimen (type of toothbrush, duration, and method of brushing). Tables 1 and 2 indicate that total bacterial counts were significantly higher at W0 than those in the remaining time periods investigated and clinical parameters of interest (GI and PI) were found to be statistically significant (*p* < 0.005). Therefore, the selection of toothpastes with superior plaque removal properties may be fundamental for the maintenance of patients' oral health. Other studies have reported that regrowth of dental plaque can be arrested by proper oral hygiene techniques [21, 22]. To the best of the authors' knowledge, the present study addresses a critical gap in knowledge because it represents the first instance where a parallel and double-blinded randomized clinical trial is conducted to assess the antiplaque and antigingivitis efficacies of toothpastes containing oxygen and lactoferrin as active ingredients, together in the same toothpaste composite, against dental plaque regrowth and improvement of GI and PI scores.

A previously published clinical trial [23] with a similar experimental design was conducted to investigate the cost-dependent efficacies of commercially available dentifrices as a function of toothbrushing frequencies (either once or twice a day). The results reported indicated that high-cost dentifrices were associated with significantly lower (*p*=0.024) GI values (0.97 ± 0.18) when compared to low-cost toothpastes (1.09 ± 0.25) when used once a day. When these toothpastes were used twice a day, results reported have indicated that no statistically significant differences (*p* > 0.05) in GI scores could be found. The PI scores after 4 weeks of utilization of the toothpastes investigated were found to be associated with no significant differences (*p* > 0.05) independently of the cost of the dentifrice considered or technique (once or twice a day) [23]. In the present study, participants were required to brush their teeth 3 times a day for 18 weeks in order to be clinically relevant and to reflect the common oral hygiene techniques delivered to patients. It is anticipated that the experimental designed herein may have contributed to mask possible differences in efficacies between the toothpastes investigated, which can be considered a limitation of the present study.

Previous studies have had already determined that Tcs is efficient in controlling dental biofilms [24–27]. The fact that no significant differences among the efficacies of the toothpastes investigated could be detected in the present study could also be related with the low number of participants per group (*n* = 18) that remained compliant with the study's guidelines at the end of the 18-week follow-up period. The small sample assigned to the intervention groups is another potential limitation of the present study. The subjects enrolled in the present study were strongly motivated to improve their oral hygiene by the prospect of oral exams and the knowledge that they were enrolled in a clinical study to identify the effects of toothpaste. This observation may be attributed to a possible Hawthorne effect, also noticed in other studies [28, 29]. Even then, the results of the present double-blinded, controlled, and randomized clinical trial have shown that dentifrices containing oxygen and lactoferrin displayed comparable efficacy in controlling dental plaque and improving clinical gingivitis as Tcs-containing dentifrices.

## 5. Conclusion

The present study demonstrated that toothpastes containing active oxygen and lactoferrin have comparable antiplaque and antigingivitis efficacies with triclosan-containing toothpastes.

## Figures and Tables

**Table 1:** Comparison between groups according to the gingival index at buccal and lingual aspects.

Gingival index (GI)					
Groups	Time				
	W −1 (mean ± SD)	W0 (mean ± SD)	W1 (mean ± SD)	W12 (mean ± SD)	W18 (mean ± SD)
CT	0.12 ± 0.16	0.59 ± 0.49^*∗*^	0.07 ± 0.10	0.12 ± 0.12	0.12 ± 0.12
BM	0.08 ± 0.14	0.62 ± 0.51^*∗*^	0.07 ± 0.12	0.13 ± 0.13	0.06 ± 0.07

W −1: preclinical trial; W0: established gingivitis; W1: 1^st^ evaluation after brushing for 1 week; W12: evaluation of brushing for 12 weeks; W18: evaluation of brushing for 18 weeks. For each group, ^*∗*^indicates significant difference between the times based on Tukey's test.

**Table 2:** Comparison between groups according to the plaque index at buccal and lingual aspects.

Plaque index (PI)					
Groups	Time				
	W −1 (mean ± SD)	W0 (mean ± SD)	W1 (mean ± SD)	W12 (mean ± SD)	W18 (mean ± SD)
CT	3.37 ± 0.55	4.09 ± 0.55^*∗*^	3.00 ± 0.48	3.16 ± 0.46	3.11 ± 0.51
BM	3.45 ± 0.63	4.16 ± 0.48^*∗*^	3.11 ± 0.63	3.12 ± 0.71	2.94 ± 0.44

W −1: preclinical trial; W0: established gingivitis; W1: 1^st^ evaluation after brushing for 1 week; W12: evaluation of brushing for 12 weeks; W18: evaluation of brushing for 18 weeks. For each group, ^*∗*^indicates significant difference between the times based on Tukey's test.

**Table 3:** Median values (min–max) of bacterial sampling count comparing the groups of toothpaste.

Groups	Growth conditions	Time			
		W0 median (min–max)	W1 median (min–max)	W12 median (min–max)	W18 median (min–max)
CT	Aerobic	1050.0 (400.0–1900.0)	308.0 (4.03–560.0)	244.0 (16.0–312.0)	197.5 (55.0–357.0)
BM		1050.0 (0–4200.0)	152.0 (63.0–280.0)	105.0 (3.0–256.0)	62.0 (6.0–280.0)

CT	Anaerobic	4000.0 (1000.0–5200.0)	428.0 (136.0–5000.0)	840 (392.0–1104.0)	352.0 (96.0–702.0)
BM		2480.0 (0.0–5000.0)	344.0 (108.0–536.0)	492.0 (91.0–1200.0)	206.0 (60.0–605.0)

## Data Availability

The data used to support the findings of this study are available from the corresponding author upon request.

## Additional Points

Active oxygen and lactoferrin toothpaste has antiplaque activity and is as efficient as triclosan toothpaste in reducing gingival inflammation.
